# A Cross-Species Single-Cell Atlas Reveals Conserved Regulatory Networks and Candidate Hearing Loss Genes in the Cochlea

**DOI:** 10.3390/genes17040438

**Published:** 2026-04-10

**Authors:** Hui Cheng, Fandi Ai, Wan Hua, Fengxiao Bu

**Affiliations:** Department of Oto-Rhino-Laryngology, Institute of Rare Diseases, West China Hospital of Sichuan University, Chengdu 610000, China; chenghui@wchscu.cn (H.C.); aifandi_1996@163.com (F.A.); huawan825@wchscu.cn (W.H.)

**Keywords:** single-cell RNA sequencing, hearing loss, cross-species comparison, transcriptional regulation

## Abstract

**Background**: The cochlea is a specialized sensory organ essential for hearing. To elucidate its cellular and molecular architecture and prioritize candidate genes associated with hearing loss (HL), we constructed a cross-species single-cell transcriptomic atlas of human fetal and postnatal mouse cochleae. **Methods**: We integrated single-cell and single-nucleus RNA sequencing datasets from human fetal cochleae and postnatal mouse cochleae to build a comprehensive cross-species single-cell transcriptomic atlas. Cell-type annotation, transcriptional regulator analysis, intercellular communication, and disease phenotypes were performed to dissect the cochlear cellular landscape, regulatory programs, and potential HL gene candidates. **Results**: A total of 19 major cochlear cell types were identified in both species, with conserved cellular composition and transcriptional programs. Comparative analysis revealed strong transcriptional conservation between matched human and mouse cell types, particularly in supporting, schwann cells and hair cells. Cell–cell communication analysis revealed conserved signaling pathways, including the BDNF-NTRK2 axis, potentially involved in cochlear development and auditory function. Regulatory network inference uncovered conserved and previously undercharacterized transcription factors, such as *SKOR1*, *RFX2*, and *PAX2*, predicted to be associated with hair cell identity and function. We further defined a conserved gene module of 3138 hair cell-enriched genes, from which 24 candidate HL-associated genes (e.g., *ATP8B1*, *BDNF*, and *SOD1*) were prioritized through integration with human disease databases and mouse auditory phenotype annotations. **Conclusions**: This study provides a high-resolution cross-species cochlear atlas, revealing conserved molecular programs and candidate HL-associated genes, offering valuable insights into auditory biology and potential avenues for further investigation.

## 1. Introduction

Hearing plays a vital role in social communication, yet over 5% of the global population (or 430 million people) suffers from disabling hearing loss (HL) at present [1]. Therefore, elucidating the mechanism of hearing loss is essential for the treatment and prevention of deafness [2]. The mammalian cochlea is a highly specialized sensory organ responsible for converting sound waves into electrical signals, a process essential for hearing [3]. This intricate function relies on the coordinated activity of numerous cell types within the auditory sensory epithelium, known as the organ of Corti. This structure comprises mechanosensory hair cells (HCs) and a diverse array of supporting cells (SCs), forming a highly heterogeneous and spatially organized tissue [4]. Damage to cochlear HCs, caused by genetic mutations, environmental insults such as noise exposure or ototoxic drugs, or progressive degeneration due to aging, is a leading cause of irreversible sensorineural hearing loss [5]. Unlike non-mammalian vertebrates, mammals lack the capacity to regenerate lost HCs, making hearing loss largely permanent once damage occurs [6,7,8,9]. Therefore, understanding the molecular underpinnings of cochlear development, cell type specification, and tissue maintenance is essential for elucidating the mechanisms of auditory dysfunction and for developing therapeutic strategies, including regenerative approaches and gene therapies.

To achieve this, animal models, particularly the mouse, have been indispensable in auditory research [10]. Owing to their genetic tractability, well-characterized developmental timelines, and overall anatomical similarity to humans, mice have enabled the discovery of essential molecular regulators of cochlear development. These include transcription factors, such as *Atoh1*, *Pou4f3*, *Barhl1*, *Rfx1*, *Rfx3*, *Insm1*, *Ikzf2*, and *Tbx2* [11,12,13,14,15,16], and signaling pathways such as Notch, Wnt, and FGF [17,18,19], which govern HC differentiation and cochlea patterning. However, while mouse models have provided invaluable insights, important evolutionary differences remain. Variations in cochlear cell composition, developmental timing, and gene regulatory networks between humans and mice raise critical questions about the translational applicability of murine findings to human biology [20]. Addressing these uncertainties requires a high-resolution, cross-species framework for comparing cochlear development and cellular identity.

In recent years, the emergence of single-cell and single-nucleus RNA sequencing (scRNA-seq and snRNA-seq) has enabled unprecedented profiling of cellular heterogeneity in complex tissues, including the inner ear [21]. Initial scRNA-seq studies in the mouse cochlea revealed distinct populations of HCs and SCs, novel marker genes, and lineage trajectories [22,23,24]. Subsequent efforts expanded to include other essential cochlear and vestibular structures such as spiral ganglion neurons, the stria vascularis, and the developing otic vesicle, leading to comprehensive cell atlases and deeper insights into transcriptional programs governing auditory and vestibular function [25,26,27,28,29]. Despite this progress, research on the human cochlea has lagged behind due to the difficulty of accessing high-quality tissue samples, particularly from embryonic or fetal stages [30,31]. As a result, much of our current understanding of inner ear development remains derived from animal models, and the extent to which these models faithfully recapitulate human biology remains largely unresolved.

Adding to this complexity, more than 200 genes have been implicated in hereditary hearing loss [32,33,34], yet their precise cell type-specific expression patterns within the cochlea, as well as the degree of their evolutionary conservation across species, remain partially defined. This knowledge gap impedes our ability to understand how genetic mutations disrupt auditory function at the cellular level and presents a major obstacle for designing effective, targeted interventions. A comprehensive, cross-species single-cell transcriptomic comparison is therefore timely needed to identify conserved gene expression programs, clarify species-specific differences, and critically evaluate the translational relevance of mouse models in the study of human hearing loss.

In this study, we systematically integrate and compare single-cell transcriptomic data from human and mouse cochlea across key developmental stages. Through comprehensive analyses of cell type composition, intercellular communication networks, transcription factor regulatory landscapes, and expression patterns of HL-related genes, we aim to identify conserved and species-specific features of cochlea biology. Our findings establish a valuable cross-species resource and provide critical insights into the molecular mechanisms of hearing, with direct implications for understanding the genetic basis of hearing loss and for guiding future therapeutic strategies.

## 2. Materials and Methods

### 2.1. Online Published scRNA-Seq/snRNA-Seq Data Collection

Single-cell and single-nucleus RNA sequencing (scRNA-seq and snRNA-seq) datasets of mouse and human cochlea were obtained from publicly available repositories. For the mouse, scRNA-seq data from postnatal day 8 (P8), P12, and P20 cochleae were retrieved from the gEAR portal (https://umgear.org/) [29]. For human samples, snRNA-seq datasets representing early developmental stages (fetal weeks 7.5 and 9.2) were downloaded from the Gene Expression Omnibus (GEO; Accession ID: GSE213796 [31]), while mid-to-late gestational cochlea scRNA-seq data (15, 17, and 23 weeks post-mortem) were obtained from GSE135913 [35]. When investigating the expression patterns of HL-related genes across different developmental stages of the mouse cochlea, we downloaded data from the GDC database, covering the following stages: E14, E18, P1, P8, P14, P20, P28, M1, M2, M5, M12, and M15 [33].

### 2.2. Ortholog Gene Selection

To enable cross-species transcriptional comparison between humans and mice, we download homologous gene lists from Ensemble BioMart (release 114) [36]. In BioMart, we selected the “Ensembl Genes” database and filtered for one-to-one orthologous gene pairs between Homo sapiens and Mus musculus. Genes with ambiguous mappings (e.g., one-to-many or many-to-many orthologs) were excluded. The resulting list of high-confidence one-to-one orthologs was used for subsequent integrative analyses between the two species.

### 2.3. Single-Cell Transcriptomic Data Analysis and Integration

All scRNA-seq analyses were performed using the Seurat package [37] (v5.1.0) in R (v4.3.2). The matrix of read-count data of each cell for each gene was loaded individually and converted to Seurat objects. To obtain high-quality cells, only cells with more than 200 genes and less than 8000 genes were detected, and less than 20% mitochondrial genes were used for subsequent analysis. The quality-controlled datasets were merged and integrated using canonical correlation analysis (CCA) integration through the IntegrateLayers function. This involved normalizing each dataset independently using Log-transformation with a scale factor of 10,000, identifying 2000 variable features per dataset by the variance stabilizing transform (vst) method, and selecting highly variable genes across datasets for integration. Integration anchors were computed and used to create an integrated dataset. Post-integration processing included data scaling with regression of RNA counts and principal component analysis (PCA). The first 30 PC were used for nonlinear dimensionality reduction using Uniform Manifold Approximation and Projection (UMAP). Clustering was performed using the “FindNeighbors” followed by the “FindClusters” functions. Each cell type was defined with the marker genes reported in previously published literature [29,31,38].

To quantitatively evaluate the effectiveness of data integration across different platforms and species, we applied the Local Inverse Simpson’s Index (LISI) [39] to the integrated dataset. The iLISI scores (Supplementary Figure S1A), which assess batch mixing, were consistently greater than 1 across datasets, indicating effective integration of cells from different sources. In contrast, cLISI scores (Supplementary Figure S1B), which measure cell type separation, were tightly centered around 1 for all cell populations. This indicates that cells of the same type remained well-clustered after integration, demonstrating that biological identity was preserved.

### 2.4. scRNA-Seq Differential Gene Expression and Enrichment Analysis

To identify signature genes of each cell types, functions “FindAllMarkers” (min.pct = 0.25, logfc.threshold = 0.25) and “FindMarkers” in Seurat were used. The function “FindMarkers” was used for identification of signature genes by comparing the cell type of interest to another specific group of cells. To elucidate the distinct expression profiles of the identified genes at the single-cell level, the “scRNAtoolVis” package (available at https://github.com/junjunlab/scRNAtoolVis, accessed on 21 March 2025) was utilized, providing a graphical interface that enabled precise and clear visualization of gene expression patterns. The “clusterProfiler” package [40] (v4.9.5) was utilized to conduct Gene Ontology (GO) and Kyoto Encyclopedia of Genes and Genomes (KEGG) analysis. GO or KEGG enrichment level was evaluated by adjusted *p*-values, and multiple test adjustment was conducted using the Benjamini–Hochberg (BH) method. Spearman’s correlation analysis was performed using R software.

### 2.5. Cell–Cell Communication Analysis

Cell–cell communication analysis based on the expression of known ligand–receptor pairs in different cell types was performed using the CellChat R package (v2.2.0) [41]. To ensure cross-species comparability, only one-to-one orthologous gene pairs between human and mice were retained, and cell annotation information and raw count expression matrix were exported from Seurat. CellChat objects for human and mouse samples were separately created, with comparative analysis introduced to uncover key ligand–receptor pairs and signaling pathways between two groups and to detect and visualize cell-state-specific cell–cell interactions. The signaling information were obtained from ligand–receptor databases (CellChatDB.human and CellChatDB.mouse). Following the official procedure, the standardized counts were inputted into CellChat, and standard preprocessing steps were performed, including functions with standard parameter settings, such as identifyOverExpressedGenes, identifyOverExpressedInteractions, and projectData. Subsequently, the computeCommunProb, computeCommunProbPathway, and aggregateNet functions were employed to calculate the strength of information flow and communication probability between different cell groups for each ligand–receptor pair. The visualization methods utilized include netVisual_circle, netVisual_bubble, netVisual_aggregate (with the layout set to circle), and plotGeneExpression.

### 2.6. Single-Cell Transcription Factor Analysis

We used the SCENIC R package [42] (v1.2.4) to infer transcription factor regulatory networks from human and mouse samples. We followed the official SCENIC guidelines and default parameters to standardize the analysis process. The SCENIC analysis consisted of four main steps. In the first step, the grnboost2 algorithm was employed to identify and filter genes co-expressed with transcription factors (TFs). The second step involved using RcisTarget (v1.26.0) to find significantly expressed target genes, then performing a significant motif enrichment analysis for each co-expressed module. In the third step, the regulon activity score is calculated to quantify the activity of regulons in each cell. Each regulon represents a TF along with its direct target genes. Finally, for each cell subpopulation, the key regulons with high RSSs are predicted by an entropy-based strategy. RSS represents the activity of regulons in cell subpopulations. Cytoscape (v3.5.1) [43] was used to portray the regulatory network connecting transcription factors and their target genes.

### 2.7. Conserved Gene Expression Programs Identification

A gene was considered expressed in a species if it was detected with a UMI count ≥1 in at least one hair cell in that species. We intersected the lists of expressed genes from human and mouse hair cells and identified a total of 12,043 commonly expressed genes for subsequent analysis. For each species, we calculated the average expression of each gene across all hair cells. To compute the percentile values of genes in hair cells, we ranked the 12,043 shared genes by their average expression levels from low to high, and then divided the rank of each gene by the total number of genes. Using this approach, we generated a percentile matrix for hair cells in both human and mouse. Hierarchical clustering was then applied to the percentile matrix to identify gene clusters with conserved expression patterns. The clustered genes were ranked by average percentile values, and the top two clusters were selected as representative conserved gene expression programs enriched in cochlear hair cells.

## 3. Results

### 3.1. Generation of Single-Cell Atlas of Human and Mouse Cochlea

**Figure 1:**
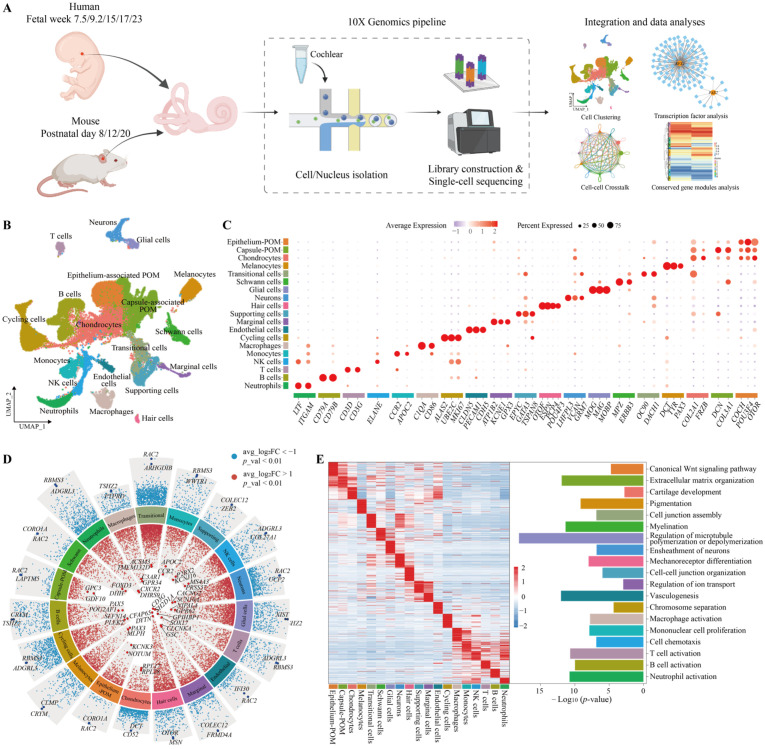
Generation of the human and mouse inner ear single-cell atlas. (**A**) Comprehensive study design for the integration of publicly available scRNA-seq/snRNA-seq datasets from human and mouse cochlea. The inset figures in the diagram were created using Biorender.com. (**B**) UMAP plot of the integrated human and mouse inner ear datasets with cell type annotations. (**C**) Expression of canonical cell type-specific marker genes for different cell types. (**D**) Volcano plot showing the differential expression of markers in the distinct cell types. Significantly upregulated genes are colored in red, and significantly downregulated genes are colored in blue, Differential expression significance was determined using a threshold of *p*-value < 0.01 and |avg_log_2_FC| > 1. (**E**) Left: heatmap showing row z-score expression signatures of top 50 cell type-specific genes. Right: representative Gene Ontology (GO) terms for top 50 genes.

To comprehensively characterize the cochlea cellular landscape across different developmental stages, we retrieved snRNA-seq and scRNA-seq datasets of human and mouse samples from public repositories. For human samples, we retrieved otic tissues at five distinct developmental stages, including fetal week (FW) 7.5, FW9.2, FW15, FW17, and FW23. In parallel, we analyzed mouse cochlea tissues at postnatal day 8 (P8), P12, and P20, corresponding to key stages of postnatal maturation (Figure 1A). The details of the three datasets enrolled in this study are listed in Supplementary Table S1. After quality control and filtering, high-confidence one-to-one orthologous genes were selected for subsequent integration analysis across the two species. Dimensionality reduction was performed using PCA followed by UMAP. We identified distinct cell populations, including mesenchymal cells, various epithelial cell types, hair cells, neurons, glial cells, endothelial cells, Schwann cells, immune cells, marginal cells, and melanocytes, totaling 19 major cell types (Figure 1B). The primary marker genes used for the annotation of each cell type are listed in Figure 1C.

To further delineate the molecular characteristics of each cell type, we applied the FindAllMarkers function to identify cell type-specific marker genes across the different clusters. The top 50 differentially expressed genes (DEGs) in each cluster are listed in Supplementary Table S2. For instance, *RPL28*, *EGFL7*, *ERMN*, *OTOF*, *IGF1*, *GPNMB*, *NRXN1*, *MPZ*, and *EPCAM* were the most significant DEG in chondrocytes, endothelial cells, glial cells, hair cells, marginal cells, melanocytes, neurons, schwann cells and supporting cells based on adjusted *p*-value. Subsequently, we employed the jjvolcano function from the scRNAtoolVis package to visualize the marker genes, emphasizing the top two most significantly upregulated and downregulated genes for each cell subtype (Figure 1D). Through functional enrichment analysis of the top 50 marker genes for each cell type, we mapped the distinct biological functions associated with different cell populations (Figure 1E). For example, mechanoreceptor differentiation was enriched in hair cells, the ensheathment of neurons was associated with neurons, regulation of ion transport was linked to marginal cells, and vasculogenesis was enriched in cochlea endothelial cells, among others. These functional enrichments highlight biological processes crucial for auditory function. Dysregulation in these functions may underlie sensorineural hearing loss.

### 3.2. Similarities Between Human and Mouse Cochlea

**Figure 2:**
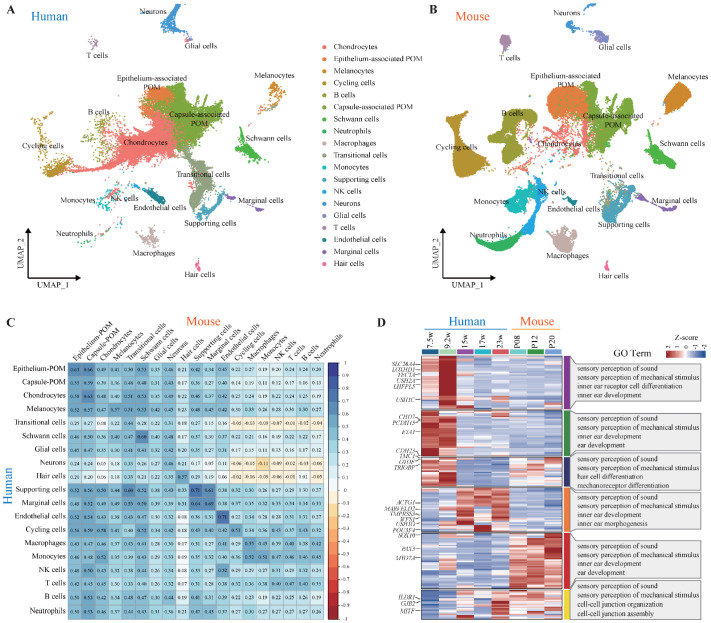
Alignment and comparison of human and mouse datasets. (**A**) UMAP plot of the integrated human inner ear datasets with cell type annotations. (**B**) UMAP plot of the integrated mouse inner ear datasets with cell type annotations. (**C**) Spearman correlation matrix of inner ear cell transcriptomes between human and mouse. Distinct correlation patterns were observed among different cell types. (**D**) The distribution of the 201 HL genes in different developmental stages of the human and mouse cochlea. The left heatmap compares the expression profiles of these genes in human and mouse. The right panels summarize the GO terms, providing insights into the biological functions associated with each cluster.

To better understand the developmental similarities and differences between human and mouse cochlea cells, we performed a comparative analysis using high-confidence one-to-one orthologous genes to evaluate cell type composition across species. Overall, the human dataset exhibited a cellular architecture highly similar to that of the mouse cochlea. We identified comparable populations of mesenchymal, chondral, epithelial, neural crest-derived, and developing hair cells in both species (Figure 2A,B). In the early developmental stages of the human cochlea, chondrocytes and a large cluster of periotic mesenchyme (POM) cells were predominant. Notably, chondrocytes were mainly detected in FW7.5 inner ear samples, where the otic vesicle was not dissected during sample preparation [31]. The epithelial compartment of the human cochlea showed remarkable similarity to that of the mouse [22,44], with distinct cell clusters corresponding to key epithelial subtypes. Specifically, we identified populations that matched marginal cells, hair cells of the organ of Corti, and various types of supporting cells. In mouse, mature supporting cells and hair cells gradually emerged between P8 and P20, consistent with the known timeline for the onset of auditory function (Supplementary Figure S2). Despite differences in developmental timing and technical limitations in capturing human cochlea samples, we did not observe major discrepancies in the transcriptional lineage between mouse and human cochleae.

To evaluate transcriptomic conservation between species, we calculated a Spearman correlation matrix based on matched inner ear cell types from humans and mice. Interestingly, different cell types exhibited varying degrees of cross-species correlation. Hair cells showed moderate correlation (R^2^ = 0.57), whereas supporting cells displayed a higher correlation (R^2^ = 0.71), suggesting that certain cell types maintain more conserved transcriptional programs across evolution (Figure 2C). We further compared the expression patterns of 201 published HL-related genes between humans and mice [33]. By analyzing gene expression across different developmental stages, we clustered the 201 HL genes into six distinct groups. We further highlighted the top 25 genes frequently diagnosed in Chinese Deafness Genetics Consortium (CDGC) patients. Among them, *SLC26A4*, *TECTA*, *CHD7*, *EYA1* and *USH2A* exhibited high expression during FW7.5 and FW9.2 in human embryonic stage, suggesting their involvement in early inner ear development. In contrast, genes such as *PAX3*, *POU3F4*, *MYO7A*, and *MITF* were predominantly expressed postnatally in mouse, implying a role in maintaining hearing function during aging (Figure 2D). Regarding cell type-specific expression, several genes, including *USH1G*, *USH2A*, *CDH23*, *OTOF*, *TMC1*, *LOXHD1*, *LHFPL5*, and *MYO7A*, showed enriched expression in hair cells, highlighting their essential roles in inner ear development. Genes primarily expressed in supporting cells, such as *GJB2*, *ILDR1*, and *SLC26A4*, are crucial for the maintenance of auditory function. Other genes, including *ACTG1*, *POU3F4*, and *SOX10*, were expressed across various other cell types, indicating broader biological functions (Supplementary Figure S3).

To further assess the robustness of cross-species comparisons, we expanded the mouse dataset by incorporating additional developmental stages (E14, E18, and P1) [33], thereby covering a broader window from embryonic development to early postnatal maturation. After integration with the human datasets, the overall cellular landscape remained largely consistent, with major cochlear cell types clearly resolved and well aligned across species (Supplementary Figure S4A,B). Differential expression analysis also identified similar sets of cell type-specific marker genes, suggesting that the molecular identities of major cell populations are stable across the extended developmental range (Supplementary Figure S4C). Cross-species correlation analysis further demonstrated that the global transcriptional relationships between corresponding cell types were largely preserved (Supplementary Figure S4D). While modest variations in correlation strength were observed, for example, a slight increase in correlations among hair cells and a mild decrease among supporting cells, these differences may reflect changes in cell composition and developmental stage distribution after including additional samples. Overall, these results indicate that our cross-species comparisons still maintain a certain level of reliability even when more developmental stages are included.

### 3.3. Estimation of Cell–Cell Communication in Human and Mouse Cochlea

**Figure 3:**
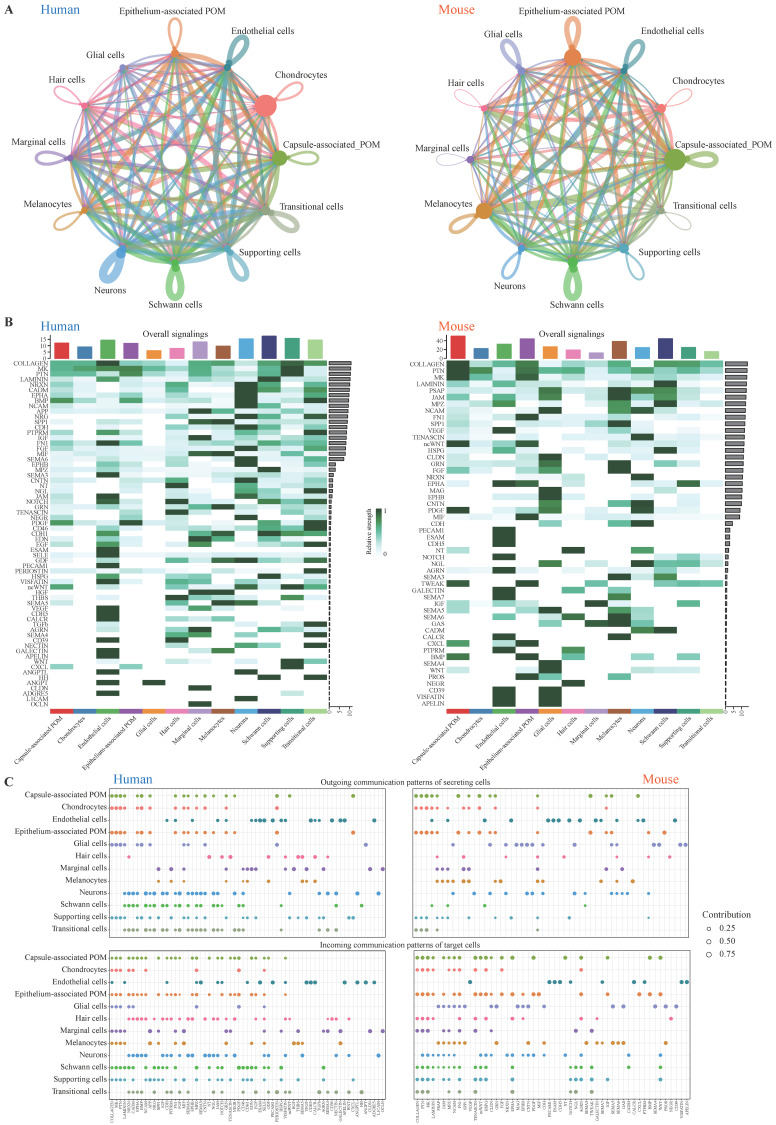
Cell–cell communication in human and mouse cochlea. (**A**) Circle plots displaying putative ligand–receptor interactions, with the width of edges representing the strength of the communication. Cell type was represented by colored node, of which the size was directly proportional to sum of receptor–ligand pairs between this node and all other nodes. (**B**) Heatmap showing the summary of the signaling pathways that contribute to overall (outgoing and incoming) communication. The color bar represents the relative signaling strength of a signaling pathway across cell types. The bars indicate the sum of the signaling strength of each cell type or pathway. (**C**) The dot plot showing the outgoing signaling patterns of secreting cells and incoming signaling patterns of targeting cells in human and mouse cochlea. The dot size is proportional to the contribution score computed from pattern recognition analysis. A higher contribution score implies the signaling pathway is more enriched in the corresponding cell group. The color of the dots represents different cell types.

To identify conserved and species-specific cell–cell communication patterns between human and mouse cochleae, we employed the CellChat R package to construct ligand–receptor-mediated signaling networks for each species. This analysis revealed extensive and dynamic intercellular communication networks within the cochlea. Overall, the topology of the signaling network among cell sources was highly similar between the two species. In terms of interaction strength between cell populations, endothelial cells, periotic mesenchyme (POM) cells, and Schwann cells emerged as major contributors to the communication network in both human and mouse cochlea, underscoring their key roles in mediating coordination within cochlear tissues (Figure 3A). Further analysis of the number of signaling pathways indicated that in the human cochlea, endothelial cells, neurons, Schwann cells, and supporting cells exhibited higher levels of total signaling activity, indicating their dominant roles in cochlea communication networks. In contrast, mouse cochlear endothelial cells and neurons demonstrated reduced signaling activity, particularly marginal cells, which may be attributable to differences in cell abundance or the developmental stage. Notably, COLLAGEN, MK, PTN, LAMININ, NCAM, and SPP1 were overall highly expressed signaling pathways in both species, many of which have been implicated in extracellular matrix organization, neural connectivity, and tissue integrity, processes closely tied to auditory function and hearing preservation (Figure 3B and Figure S5).

Moreover, to identify the key incoming and outgoing signals associated with cochlea cell subpopulations, the ligand receptor network was quantitatively measured using CellChat to predict its key incoming and outgoing signals by utilizing pattern recognition approaches. For example, in human and mouse cochlea, each type of cell could be the secreting cell (signaling senders), which could release different cytokines or ligands, and each cell type could also be the targeting cells (signaling receivers) when the receptors on these cells are targeted by the ligands released from the same types of cells or other cells. The ligand–receptor-mediated communications among different cell types should contribute to the development of cochlea. Our analysis revealed that diverse cochlear cell types, such as endothelial cells, glial cells, neurons, and supporting cells, exhibit distinct signaling roles across species. These directional interactions likely contribute to the establishment and maintenance of cochlear architecture and function (Figure 3C). Given the critical role of hair cells in auditory transduction [45], we further investigated ligand–receptor pairs mediating interactions between hair cells and other cochlear cell types. In the human cochlea, the NRXN3-NLGN1 pathway (Commun. Prob. = 0.16) was predominant in communication between hair cells and both Schwann cells and transitional cells, whereas the CADM1-CADM1 pathway (Commun. Prob. = 0.13) was primarily involved in interactions between hair cells and neurons. In contrast, the mouse cochlea exhibited prominent activity of the Psap-Gpr37 pathway (Commun. Prob. = 0.19), which mediated interactions between hair cells and glial cells as well as melanocytes, suggesting a potential role in cochlear homeostasis and neuroprotection through glia-associated signaling. Notably, the BDNF-NTRK2 (Human. Commun. Prob. = 0.10, Mouse. Commun. Prob. = 0.21) pathway showed significant activity in mediating interactions between hair cells and spiral ganglion neurons in both species, consistent with findings reported by Ma et al. [46], as well as highlighting a conserved neurotrophic signaling mechanism during cochlear development (Supplementary Figure S6 and Supplementary Table S3). These results underscore the significance of potential ligand–receptor networks, particularly the BDNF-NTRK2 (Bdnf-Ntrk2) communication pathways, which may play a crucial role in maintaining auditory function.

### 3.4. Conservation of Hair Cell Regulomes in Human and Mouse Cochlea

**Figure 4:**
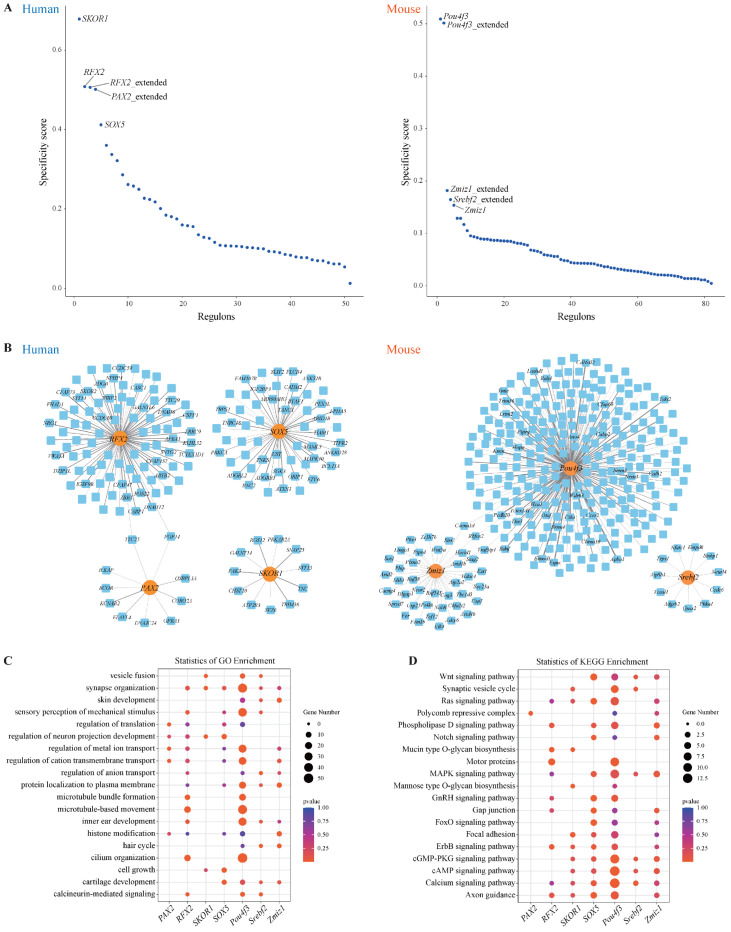
TFs showing regulon activities in hair cells. (**A**) Regulon specificity score in hair cells with top 5 specific TFs labeled. (**B**) Genetic regulatory network constructed by specific TFs and predicted target genes in hair cells across human and mouse cochlea. Orange nodes represent the regulators, and light blue nodes represent corresponding target genes. Edge width is proportional to weight of regulation. For nodes with a large number of TF-target genes, the top 30 genes with the largest weight values are presented. (**C**) GO enrichment related to cellular functions of predicted target genes of top 5 TFs in hair cells. The top 3 enriched GO terms for each TF were selected for display. Dot color represents significance level of enrichment analysis and dot size is count of target genes classified in GO terms. (**D**) KEGG enrichment of predicted target genes of top 5 TFs in hair cells. The top 3 enriched pathways for each TF were selected for display. Dot color represents significance level of enrichment analysis and dot size is count of target genes classified in pathways. *p*-values were calculated using hypergeometric test.

Given that transcriptional dysregulation of hair cell development and function is a major cause of hearing loss, we systematically investigated key transcriptional regulators in cochlear hair cells of both humans and mice using the SCENIC R package [42]. This analysis revealed 2771 transcription factor (TF)–target gene interactions in human hair cells and 4439 such interactions in mouse hair cells (Supplementary Table S4). The regulators that were specific to the identified cells were arranged from large to small according to the regulon specificity score (RSS). In the human hair cells, the top five TFs included *SKOR1*, *RFX2*, *PAX2*, and *SOX5*, while in the mouse hair cells, *Pou4f3*, *Zmiz1*, and *Srebf2* were among the most specific (Figure 4A). Several well-established transcription factors involved in cochlear hair cell development, including *POU4F3*, *ATOH1*, and *BARHL1*, were also identified, supporting the validity of the SCENIC-based inference. Notably, our analysis highlighted several previously undercharacterized transcription factors, such as *SKOR1*, *RFX2*, and *PAX2*, as putative regulators whose regulons are enriched in hair cells and are predicted to be associated with hair cell identity and function. These predictions suggest that these TFs may participate in transcriptional programs relevant to hair cell development and maintenance, although functional validation will be required to confirm their regulatory roles. Moreover, in addition to the *POU4F3* gene, *SKOR1* also shows a strong specific expression in hair cells (Supplementary Figure S7). We further constructed TF-target gene regulatory networks, illustrating the connections between top-ranked TFs and their predicted downstream targets. These networks illustrated not only direct regulatory relationships but also potential co-regulation of targets by multiple TFs, suggesting a convergence of transcriptional control (Figure 4B).

To investigate the functional roles of these TFs, we performed GO and KEGG enrichment analyses on their predicted target genes. GO analysis suggested that *Pou4f3* target genes were associated with cilium organization and inner ear development, whereas *SKOR1* target genes were enriched in terms such as regulation of neuron projection development and synapse organization (Figure 4C). KEGG pathway analysis further revealed the involvement of these TFs in key biological pathways, including the cAMP signaling pathway, calcium signaling pathway, and axon guidance (Figure 4D). Overall, our study systematically identified conserved regulators for hair cells in human and mouse cochlea, including both well-recognized and previously undercharacterized regulators.

### 3.5. Identification of Conserved Gene Modules and Candidate HL-Associated Genes in Cochlear Hair Cells

**Figure 5:**
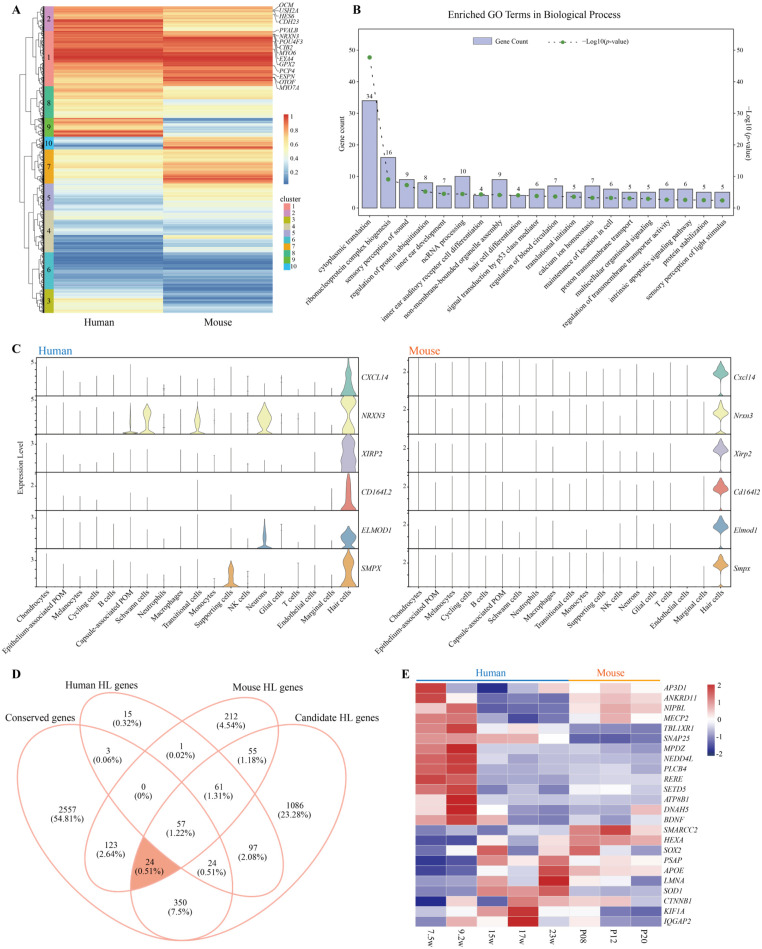
Conserved core expression programs in hair cells. (**A**) Heatmap showing clustering of orthologs in hair cells across human and mouse, with cluster index displayed on left. Each row represents one ortholog and each column represents one species. Colors represent expression levels of orthologs. (**B**) Bar plot showing significantly enriched GO terms in the set of conserved hair cell orthologs (Biological Process category). Bars represent the number of genes associated with each GO term (left y-axis), while the green dot indicates the statistical significance as −log10(*p*-value) (right y-axis). (**C**) Violin plots showing expression levels of selected conserved hair cell genes in different cochlear cell types for both human (**left**) and mouse (**right**). (**D**) The Venn diagram shows the screening process of 24 candidate HL-associated genes. Explanation: Conserved genes refer to conserved gene expression modules in hair cells, totaling 3138 genes. Human HL genes were curated from three major databases—HHL, DVD, and GDC—all of which include genes implicated in hereditary hearing loss in humans. Mouse HL genes were obtained from the MGI database, where genes linked to abnormal hearing phenotypes in mouse models were included. Candidate HL genes refer to HL-related genes identified through diseases databases, which may not yet have direct functional validation in HL. (**E**) The heatmap shows the expression distribution of 24 candidate HL-associated genes in different developmental stages of the human and mouse cochlea.

To investigate the conserved molecular signatures of hair cells across species, we identified orthologous genes with consistent expression patterns in cochlear hair cells from both humans and mice. In brief, we selected 12,043 genes that were detectably expressed in hair cells of both species and performed hierarchical clustering analysis. Among these, clusters 1 and 2 comprising 3138 genes (Figure 5A and Supplementary Table S5) exhibited conserved expression profiles across humans and mice and were therefore defined as the core conserved gene expression program of hair cells. Notably, the conserved modules included many well-known hair cell markers, such as *ESPN*, *MYO6*, *OTOF*, *PCP4*, and *POU4F3*, supporting the biological validity of our approach. To further explore the functional relevance of these conserved genes, we performed GO enrichment analysis on the top 100 genes with the highest expression percentages within the conserved modules. The results revealed strong enrichment in processes essential for hair cell identity and function, including cytoplasmic translation, sensory perception of sound, inner ear development, and hair cell differentiation (Figure 5B and Supplementary Table S6). We also examined the expression distribution of several top-ranking conserved genes (*CXCL14*, *NRXN3*, *XIRP2*, *CD164L2*, *ELMOD1* and *SMPX*) across cochlear cell types. Among these, *SMPX* is a known HL-related gene [30], while the others showed highly specific expression in hair cells, suggesting their potential as novel hair cell marker genes (Figure 5C).

To assess whether genes in the conserved modules may be involved in HL, we first removed known deafness genes cataloged in HHL (https://hereditaryhearingloss.org/), DVD [32], and GDC [33] databases. The remaining genes were then filtered based on two criteria: (1) evidence of hearing-related phenotypes in mouse (MGI database [47]), and (2) association with hearing loss in human disease-related databases, including DisGeNET [48], GenCC [49], Orphanet [50], PanelApp [51], OMIM [52], Genetic Testing Registry (GTR) [53], and HPO [54]. This analysis yielded 24 candidate HL-associated genes (Figure 5D and Supplementary Table S5), which have not been previously established as causal HL genes in humans but are supported by hearing-related phenotypes in mouse models and disease associations in human databases. GO enrichment analysis of these 24 genes revealed that *ATP8B1*, *HEXA*, *NIPBL*, and *SOD1* are involved in functions such as sensory perception of sound and mechanical stimulus, highlighting their potential roles in hearing (Supplementary Figure S8). Previous studies have reported that *ATP8B1* deficiency leads to progressive degeneration of cochlear hair cells and hearing loss [55], while *SOD1* has been implicated in age-related and noise-induced hearing loss [56]. Moreover, temporal expression analysis showed that most of these genes are highly expressed during early cochlear developmental stages in both species, suggesting critical roles in early inner ear development (Figure 5E). Together, these findings define a conserved transcriptional program underlying hair cell identity and reveal a set of candidate genes that may contribute to the pathogenesis of hearing loss across mammals.

### 3.6. An Atlas of Hearing Loss Gene Expression During Cochlear Development

To investigate the spatiotemporal expression patterns of HL-associated genes in the cochlea, we analyzed transcriptomic data across different developmental stages of the mouse cochlea, due to the limited availability of temporally resolved human cochlear samples. A total of 302 HL-related genes were curated from the HHL, DVD [32], and GDC [33] databases, as well as from the GWAS catalog for age-related hearing loss (ARHL) (https://www.ebi.ac.uk/gwas/, accessed on 16 May 2025) (Supplementary Table S7). Notably, some of these genes have not yet been extensively studied in the inner ear.

**Figure 6:**
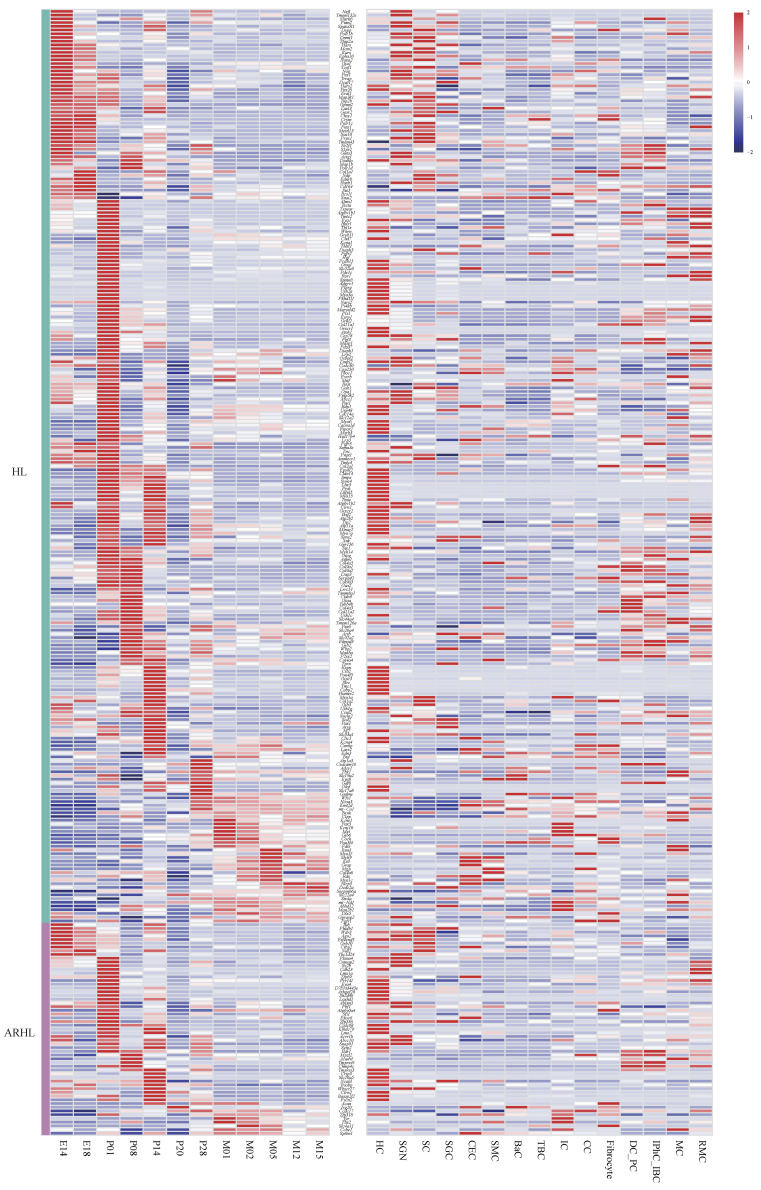
Localization of hearing loss genes. Heatmaps illustrating the expression distribution of hearing loss genes across different developmental stages and cell types in the mouse cochlea. Abbreviations: HL, Hearing loss; ARHL, Age-related hearing loss; E14: Embryonic day 14; P01: Postnatal day 1; M01: 1 month old; HC, Hair cell; SGN, Spiral ganglion neuron; SC, Schwann cell; SGC, Satellite glial cell; CEC, Capillary endothelial cell; SMC, Smooth muscle cell; BaC, Basal cell; TBC, Tympanic border cell; IC, Intermediate cell; CC, Chondrocyte; DC_PC, Deiter cell and pillar cell; IPhC_IBC, Inner phalangeal cell/inner border cell; MC, Marginal cell; RMC, cells in Reissner’s membrane.

The average expression levels of these HL genes across developmental stages and cochlear cell types are shown in Figure 6. Most HL genes exhibited higher expression levels postnatally. For instance, genes such as *Atoh1*, *Eya4*, *Gjb3*, *Kcnq1*, *Mitf*, *Pou4f3*, and *Whrn* implying potential roles in maintaining auditory function during aging. In contrast, *Actg1*, *Clrn1*, *Eral1*, and *Sox10* were highly expressed during embryonic stages, suggesting roles in early inner ear development. Although ARHL-associated genes identified by GWAS show dynamic expression patterns across developmental stages, most also exhibit a postnatal upregulation trend, such as *Acvr1b*, *Cntnap2*, *Isg20*, *Sfi1*, and *Sv2b*. This may reflect their roles in maintaining the function and homeostasis of mature auditory cells rather than in early development. As the cochlea continues to mature after birth, especially around the onset of hearing, these genes likely become more active to support auditory signaling and protect against age-related stress, highlighting their potential involvement in ARHL when dysregulated.

In terms of cell type specificity, most HL genes were enriched in hair cells and supporting cells, consistent with previous findings. For example, *Myo6*, *Myo7a*, *Myo3a*, *Cdh23*, *Pcdh15*, *Gipc3*, *Espn*, *Otof*, *Ush2a*, and *Cib2* were predominantly expressed in hair cells. *Acvr1b*, *Atp1a3*, *Map1b*, and *Spata5l1* were predominantly expressed in Spiral ganglion neuron. *Gjb2*, *Elmod3* and *Otog* exhibited highest expression in inner phalangeal cells or inner border cells. Other genes such as *Actg1*, *Aifm1*, *Pou3f4*, *Myh14*, *Snai2*, *Sox10*, and *Tbx1* were broadly expressed across multiple cochlear cell types, suggesting diverse functional roles in cochlear development and maintenance.

## 4. Discussion

In this study, we present a comprehensive single-cell transcriptomic atlas of the human and mouse cochlea across multiple developmental stages, revealing the cellular, transcriptional, and signaling landscapes that underlie auditory function. Notably, we identified a conserved gene module comprising 3138 genes specifically expressed in hair cells of both species. By integrating data from HL-associated databases and known mouse auditory phenotypes, we have screened out 24 candidate genes potentially associated with HL. These findings provide novel insights into the molecular basis of sensorineural hearing loss and highlight conserved regulatory programs in cochlear hair cells. Additionally, we mapped the expression trajectories of 302 HL-associated genes, revealing their dynamic and cell type-specific expression during cochlear maturation, particularly highlighting the postnatal upregulation of age-related hearing loss genes, which may reflect their roles in auditory function preservation during aging.

Importantly, Our SCENIC-based regulome analysis uncovered previously undercharacterized transcription factors, such as *SKOR1*, *RFX2*, and *PAX2*, which exhibited strong hair cell-specific activity in human cochlea. Notably, previous studies have demonstrated that these transcription factors are abundantly expressed in hair cells, supporting their potential functional significance [57,58,59]. The conserved activity of these transcription factors, and their association with pathways such as axon guidance and synapse organization, underscores the complex and coordinated regulation of sensory transduction machinery in the inner ear. These previously undercharacterized regulators may represent candidates for future functional studies, which could explore their roles in gene therapy or reprogramming approaches for auditory restoration.

Furthermore, our analysis of ligand–receptor interactions revealed conserved communication pathways, such as BDNF-NTRK2 signaling between HCs and spiral ganglion neurons, a pathway previously implicated in neurotrophic support of auditory neurons [45]. Interestingly, species-specific signaling interactions also emerged—for example, the Psap-Gpr37 axis in mouse cochlea—suggesting species-dependent roles in glial-neuronal communication. These findings highlight the complexity of cochlear signaling microenvironments and provide a framework for exploring cellular crosstalk in both normal development and disease contexts.

Our findings reinforce and expand upon prior work demonstrating conserved epithelial and neural crest-derived lineages in the cochlea [60,61,62]. The strong cross-species transcriptional correlations observed in supporting cells (R^2^ = 0.71), hair cells (R^2^ = 0.57), and other cochlear cell types validate the utility of mouse models in auditory research. Furthermore, the conserved expression of many HL-related genes, including *OTOF*, *MYO6*, *POU4F3*, and *GJB2*, supports previous observations of shared genetic risk factors for hereditary hearing loss. Notably, our results add value by identifying developmental timing differences in gene expression (e.g., *SLC26A4*, *EYA1*, and *POU3F4*) between human and mouse cochlea, which may help explain species-specific differences in phenotypic manifestations of deafness.

Despite the strengths of this study, several limitations should be acknowledged. First, the number of available human cochlear samples remains limited, and the temporal resolution across developmental stages is relatively low. In addition, inner and outer hair cells were analyzed jointly due to limited cell numbers, which restricts our ability to perform a comprehensive comparison of late-stage hair cell maturation between species. Second, although we integrated datasets from multiple high-confidence public resources, batch effects and technical variability across studies may still influence the observed clustering patterns and gene expression profiles. Additionally, the functional roles of the newly identified candidate genes and regulatory networks remain to be experimentally validated. Therefore, their precise contributions to auditory development and hearing loss should be interpreted with caution. Integration of spatial transcriptomics and epigenomic data in future studies could further enhance the understanding of cell type interactions and gene regulation within the cochlea [63,64].

## 5. Conclusions

In conclusion, our cross-species single-cell atlas defines conserved cellular and molecular programs of cochlear development and identifies candidate genes and regulatory factors associated with hearing loss. These findings not only validate the translational value of mouse models in auditory research but also emphasize the importance of human-specific analyses for understanding the genetic basis of hearing loss. Overall, this work has promoted our understanding of auditory biology and lay the foundation for future diagnostic and therapeutic efforts targeting hearing disorders.

## Data Availability

This study involved the analysis of publicly available datasets, which can be accessed through the following links: https://www.ncbi.nlm.nih.gov/geo/query/acc.cgi?acc=GSE213796 (accessed on 28 December 2024), https://www.ncbi.nlm.nih.gov/geo/query/acc.cgi?acc=GSE135913 (accessed on 6 January 2025), and the gEAR Portal (https://umgear.org/) in the datasets “scRNA-seq-P8, P12, P20 mouse cochleae [29] (accessed on 15 January 2025)”.

## Supplementary Materials

The following supporting information can be downloaded at: https://www.mdpi.com/article/10.3390/genes17040438/s1, Figure S1: Quantitative assessment of data integration using LISI metrics; Figure S2: UMAP plots showing the cochlea cell distribution from various ages in humans and mouse.; Figure S3: The expression distribution of the 201 HL genes in different cell types of the human and mouse cochlea; Figure S4: Generation of the human and mouse inner ear single-cell atlas; Figure S5: cell-cell communication in human and mouse cochlea as analyzed by CellChat program; Figure S6: cell-cell communication in human and mouse cochlea; Figure S7: Expression of TF genes for different cell types; Figure S8: Gene ontology (GO) enrichment analysis. Table S1: Overview of the datasets in the current study; Table S2: The top 50 DEGs in each cell cluster; Table S3: Cell-cell communication in human and mouse cochlea; Table S4: The list of TF-target pairs in hair cells from human and mouse cochlea; Table S5: Expression percentile matrix of 12,043 candidate genes; Table S6: GO term enrichment of hair cell conserved core expression program; Table S7: The annotation information of HL gene.
