# Supplementary material for: A Cross-Species Single-Cell Atlas Reveals Conserved Regulatory Networks and Candidate Hearing Loss Genes in the Cochlea
Source: Genes (Basel). 2026 Apr 10;17(4):438. doi: 10.3390/genes17040438 (PMC13115875; doi:10.3390/genes17040438)
Supplement: Supplementary file 1 [file genes-17-00438-s001.zip › Supplementary material.docx]

Supplementary Materials for

**A cross-species single-cell atlas reveals conserved regulatory networks and candidate hearing loss genes in the cochlea**

*Hui Cheng*, *Fandi Ai*, *Wan Hua*, *Fengxiao Bu*^,*^

* Corresponding author: bufengxiao@wchscu.cn (F. Bu)

**This PDF file includes:**

Figures S1 to S8

**Other Supplementary Material for this manuscript includes the following:**

Tables S1 to S7


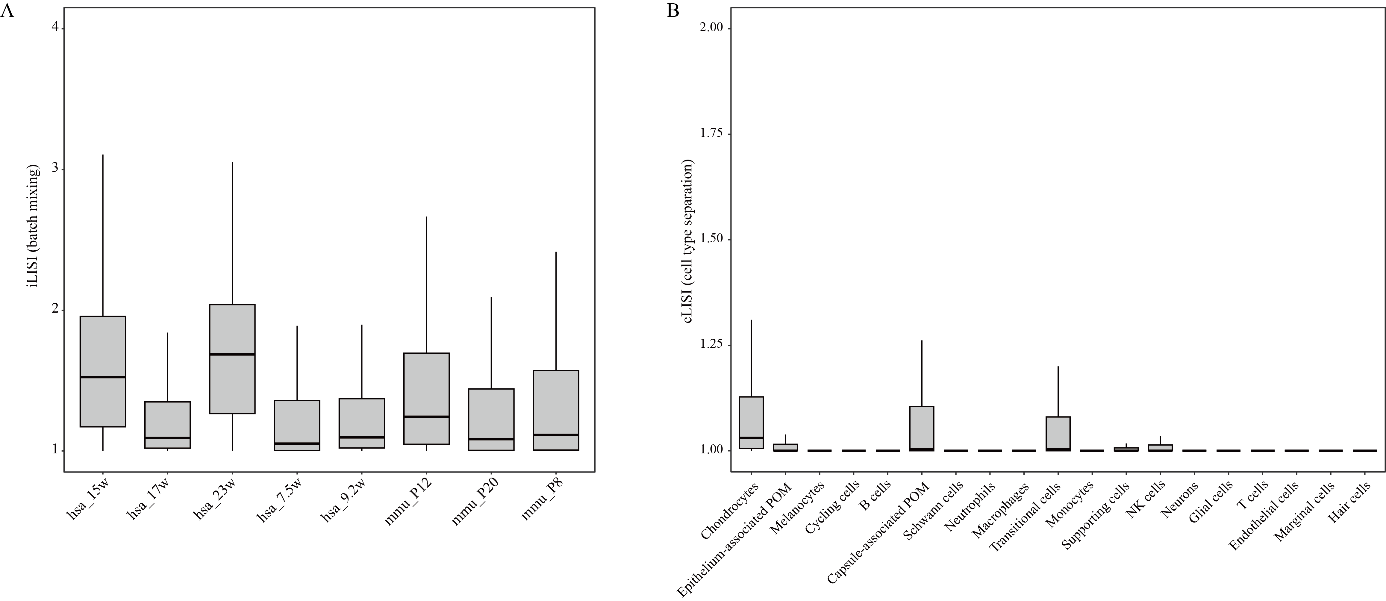


**Supplementary** **Figure S1:** Quantitative assessment of data integration using LISI metrics.

**(A)** iLISI scores indicating batch mixing across datasets. Higher values reflect improved integration. **(B)** cLISI scores indicating cell type separation. Values close to 1 suggest preservation of cell identity.


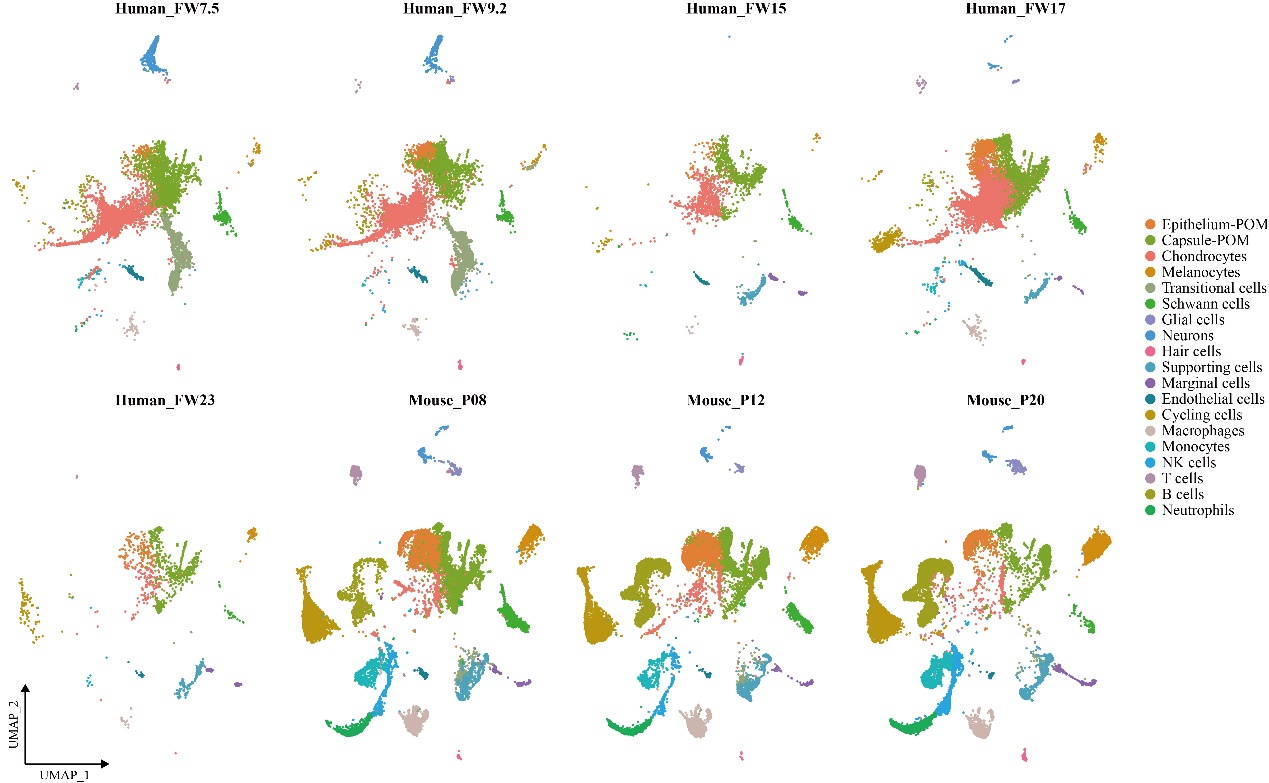


**Supplementary Figure S2:** UMAP plots showing the cochlea cell distribution from various ages in humans and mouse. FW: fetal week; P: postnatal.


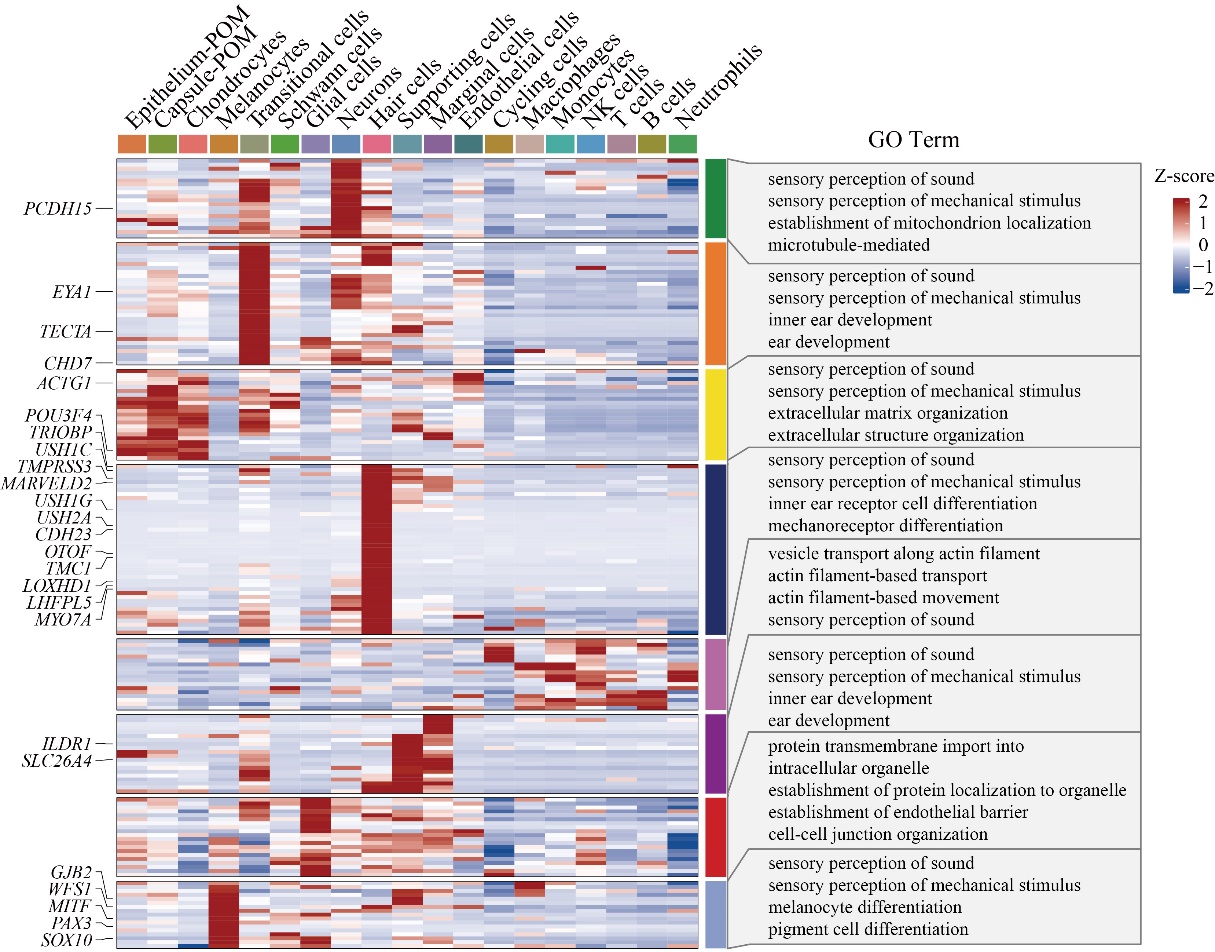


**Supplementary** **Figure S3:** The expression distribution of the 201 HL genes in different cell types of the human and mouse cochlea. The left heatmap compares the expression profiles of these genes in different cell types. The right panels summarize the GO terms, providing insights into the biological functions associated with each cluster.


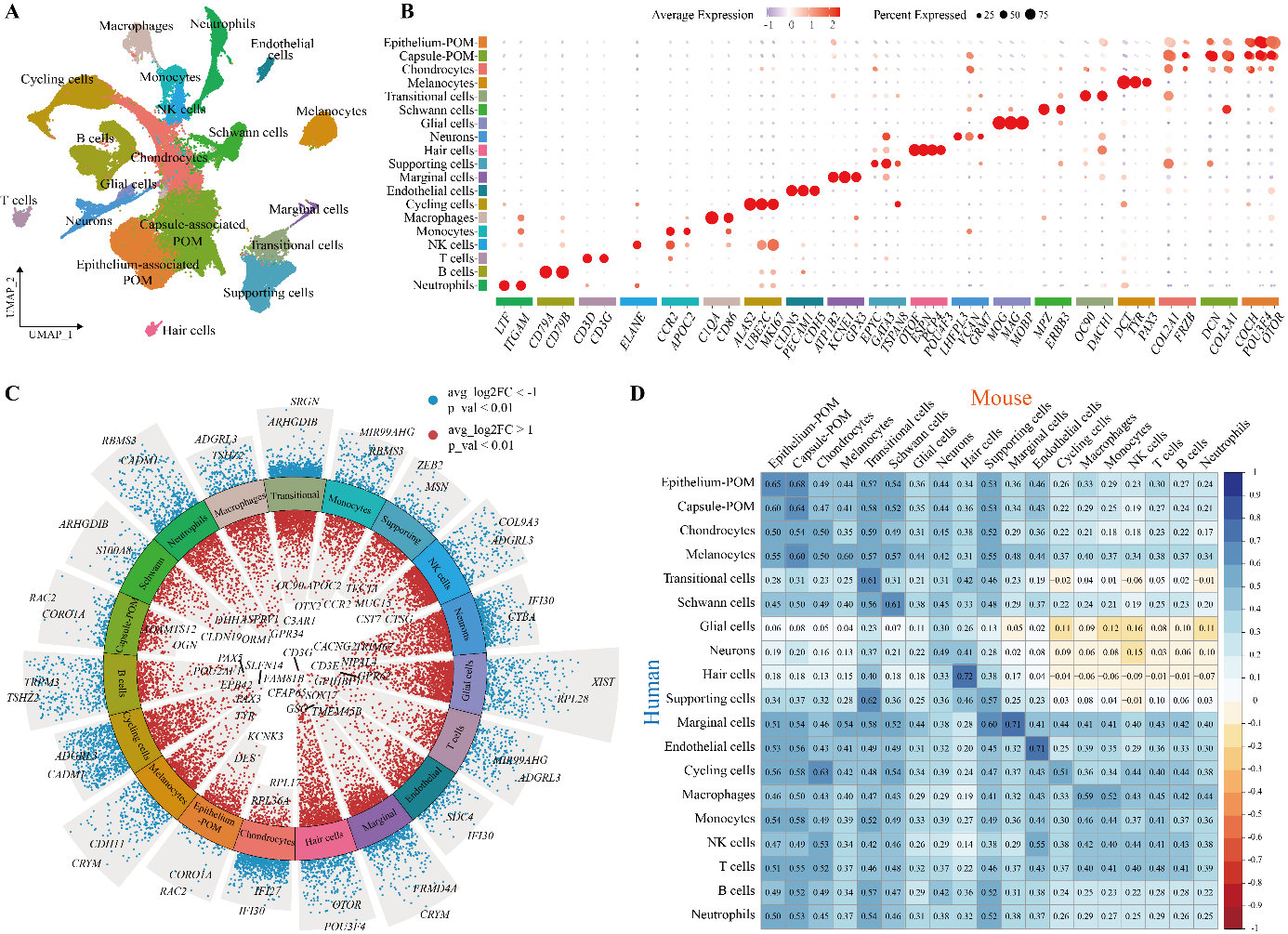


**Supplementary** **Figure S4:** Generation of the human and mouse inner ear single-cell atlas.

**(A)** UMAP plot of the integrated human (FW7.5, FW9.2, FW15, FW17, and FW23) and mouse (E14, E18, P1, P8, P12, and P20) inner ear datasets with cell type annotations. **(B)** Expression of canonical cell-type-specific marker genes for different cell types. **(C)** Volcano plot showing the differential expression of markers in the distinct cell types. Significantly upregulated genes are colored in red, and significantly downregulated genes are colored in blue, Differential expression significance was determined using a threshold of p-value < 0.01 and |avg_log2FC| > 1. **(D)** Spearman correlation matrix of inner ear cell transcriptomes between human and mouse. Distinct correlation patterns were observed among different cell types.


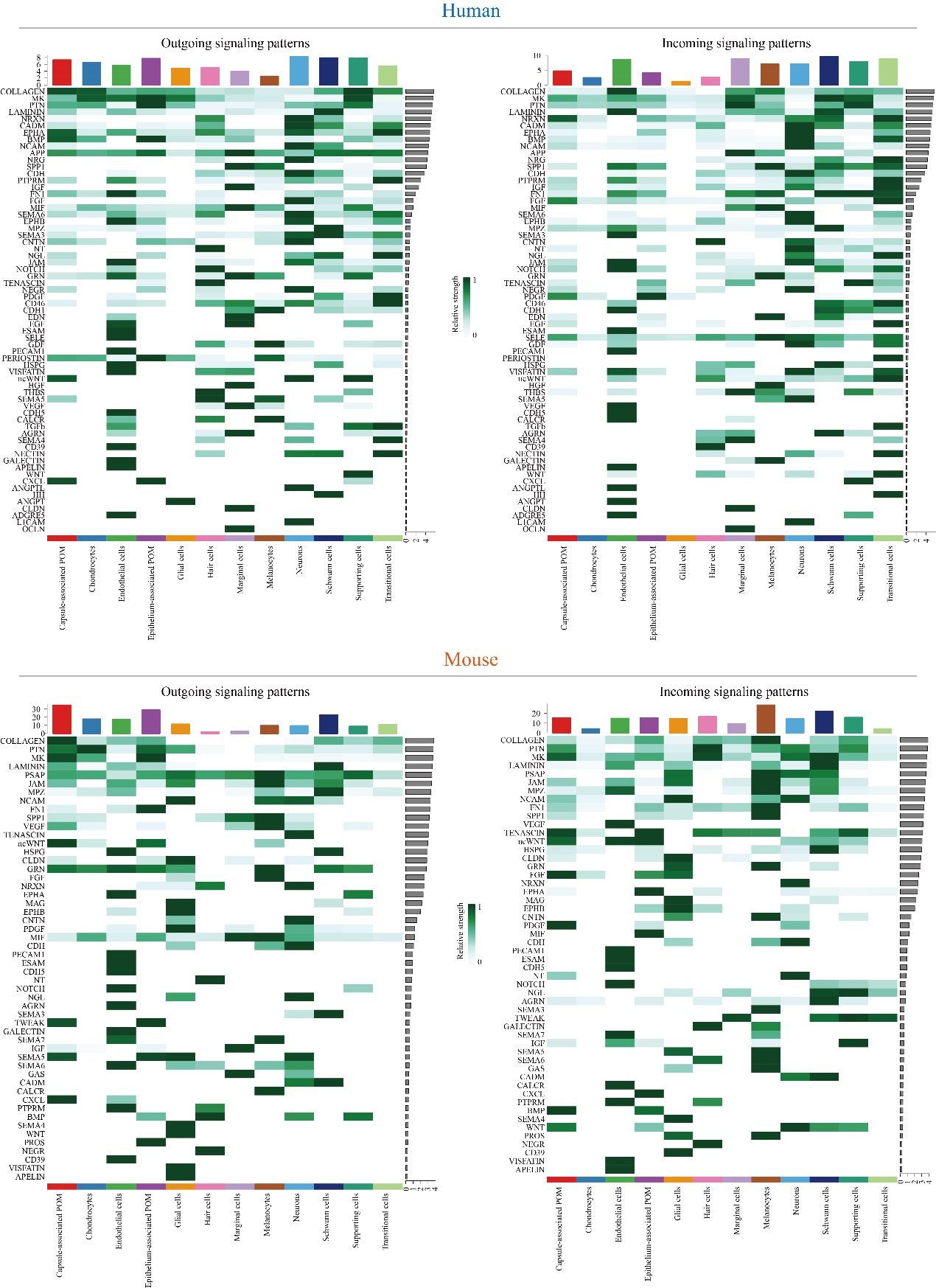


**Supplementary** **Figure S5:** cell-cell communication in human and mouse cochlea as analyzed by CellChat program. Heatmap showing the summary of the signaling pathways that contribute to outgoing or incoming communication. The color bar represents the relative signaling strength of a signaling pathway across cell types. The bars indicate the sum of the signaling strength of each cell type or pathway.


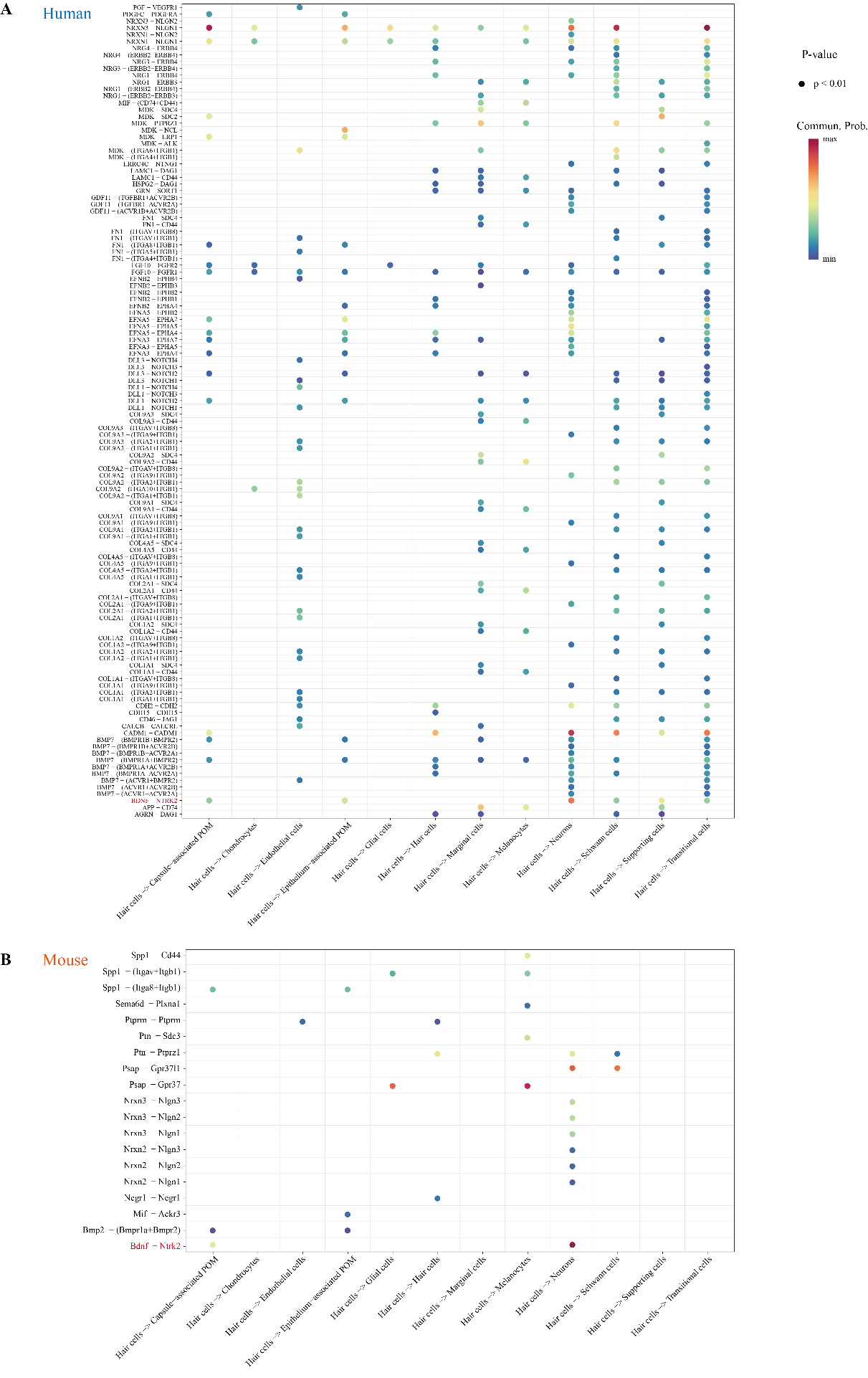


**Supplementary** **Figure S6**: cell-cell communication in human and mouse cochlea.

**(A)** The identification of ligand-receptor pairs that may interact with hair cells in human. **(B)** The identification of ligand-receptor pairs that may interact with hair cells in mouse. Bubble size represents P-value generated by the permutation test, and the color represents the possibility of interactions. Empty space means the communication probability is zero.


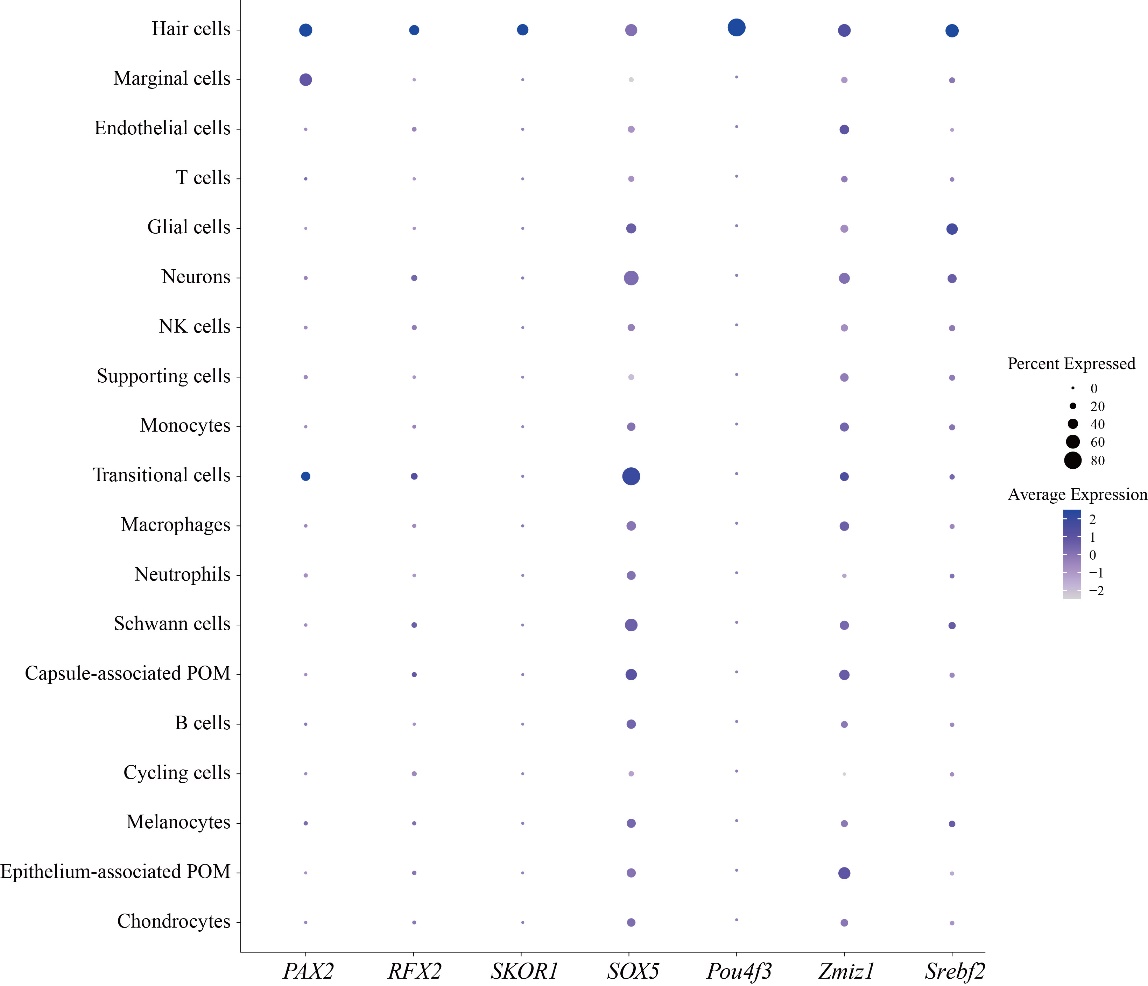


**Supplementary** **Figure S7:** Expression of TF genes for different cell types.


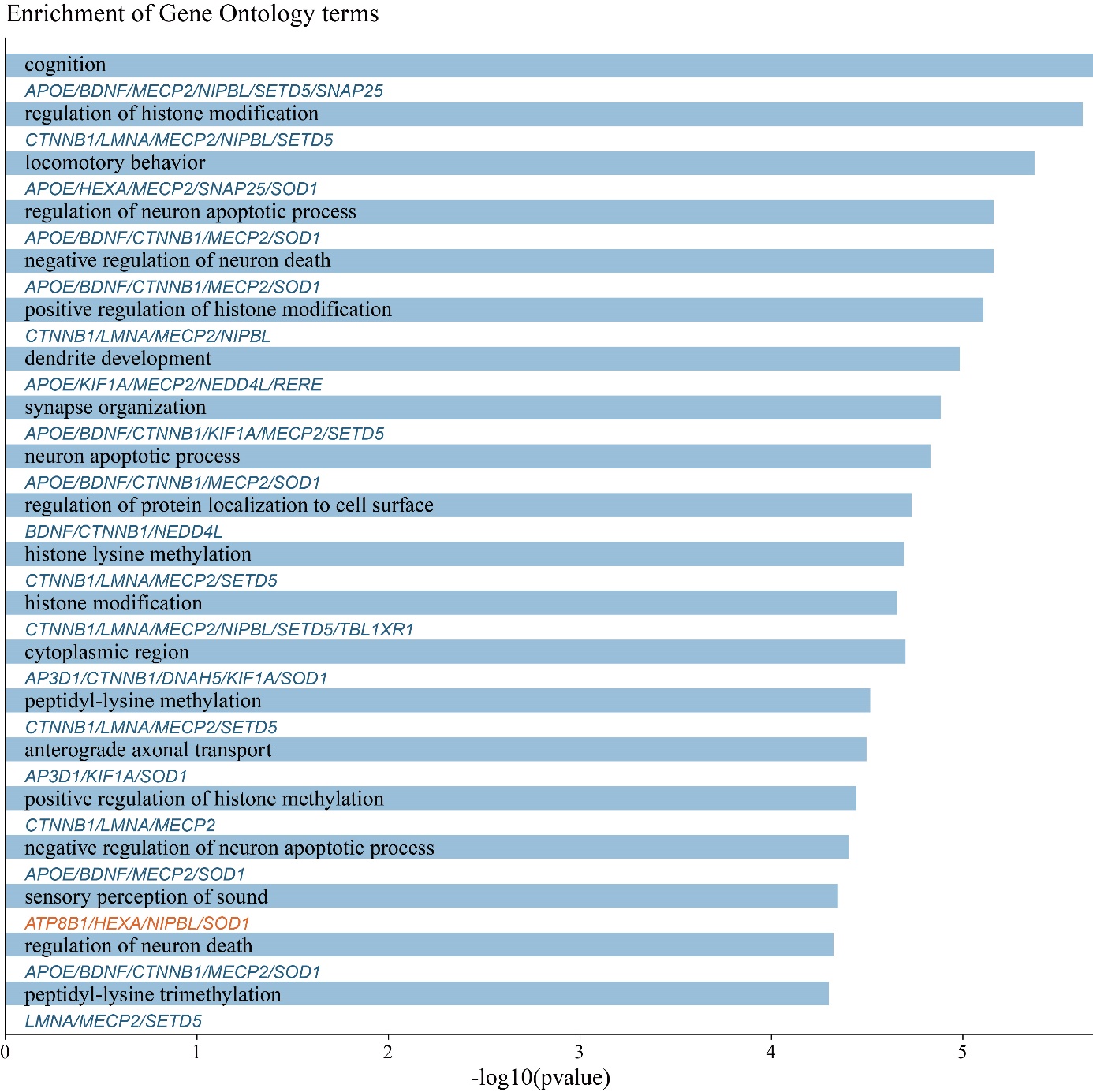


**Supplementary** **Figure S8:** Gene ontology (GO) enrichment analysis. Bar plot lists the top 20 enrichments of BP (Biological Process) category based on p-value. On the bottom, the bar plot shows the names of the GO terms. Below each GO term, the corresponding gene for that entry is displayed. Orange represents genes related to sensory perception of sound.

**Supplementary Tables:**

Supplementary Table S1: Overview of the datasets in the current study

Supplementary Table S2: The top 50 DEGs in each cell cluster

Supplementary Table S3: Cell-cell communication in human and mouse cochlea

Supplementary Table S4: The list of TF-target pairs in hair cells from human and mouse cochlea

Supplementary Table S5: Expression percentile matrix of 12,043 candidate genes

Supplementary Table S6: GO term enrichment of hair cell conserved core expression program

Supplementary Table S7: The annotation information of HL genes
